# The Agentic AI Framework (AAIF): a policy-enforced architecture for accountable and high-performance intrusion detection

**DOI:** 10.3389/frai.2026.1755696

**Published:** 2026-06-04

**Authors:** Ibrahim Adabara, Bashir Olaniyi Sadiq, Aliyu Nuhu Shuaibu, Yale Ibrahim Danjuma, Venkateswarlu Maninti, Mutebi Joe

**Affiliations:** 1 Department of Computing, Faculty of Science and Technology, Kampala International University, Kampala, Uganda; 2 Department of Electrical, Telecommunication, and Computer Engineering, Kampala International University, Kampala, Uganda

**Keywords:** accountability, agentic AI, AI governance, cybersecurity, explainable AI, intrusion detection system, NIST AI risk management framework, policy-aware AI

## Abstract

Artificial intelligence plays a central role in modern cybersecurity, yet systems optimized for detection accuracy often lack mechanisms for accountability, transparency, and policy compliance. This study proposes the Agentic AI Framework (AAIF), a policy-aware intrusion detection architecture that integrates predictive modeling with executable governance. Guided by Design Science Research, the framework combines a deep learning detection model with a governance layer aligned to the NIST AI Risk Management Framework 2.0. A key component is an interpretable Policy Engine that enforces operational and ethical constraints through a declarative YAML-based domain-specific language, ensuring that each decision is auditable and policy-compliant. The framework was evaluated on the CICIDS2017 dataset, which contains over 2.8 million network flow records across benign and malicious traffic. Results show that AAIF preserves predictive performance relative to baseline models, including Random Forest, Support Vector Machine, and Deep Neural Network, achieving a weighted *F*1-score of 0.483 and an AUROC of 0.978. At the same time, the framework achieved complete compliance under the defined policy schema, with an Ethical Compliance Rate of 1.0 and a False Escalation Rate of 0.0. The Governance Compliance Index improved from 0.947 to 0.983, demonstrating stronger alignment between system decisions and governance requirements. These findings show that policy-enforced inference can support accountable autonomy without degrading detection capability. The AAIF provides a reproducible and governance-aware approach that transforms conventional intrusion detection systems into transparent and auditable decision systems. This work establishes a practical foundation for deploying policy-aligned AI in cybersecurity environments.

## 1 Introduction

Artificial intelligence (AI) has become a central component of modern cybersecurity, supporting threat detection, vulnerability analysis, and incident response across complex digital infrastructures. Machine learning-based intrusion detection systems (IDSs) have significantly improved detection capability by identifying patterns and behaviors that are difficult to capture using static rule-based approaches (Azarudeen et al., 2024; Villegas-Ch et al., 2025). However, as these systems become more autonomous, concerns about accountability, transparency, and policy compliance have become more prominent.

Recent studies show that high-performing models often lack mechanisms for explainability, traceability, and alignment with governance principles such as fairness and responsibility (Ahangar et al., 2025; Camilleri, 2024; Cheong, 2024; Herrera-Poyatos et al., 2025; Zhang et al., 2024). This creates a gap between predictive performance and responsible deployment. In security operations centers, automated decisions such as blocking or escalating network traffic can affect critical systems and user privacy. When these decisions are not transparent or policy-aligned, they may violate institutional or regulatory requirements.

The core problem addressed in this study is that high detection accuracy does not guarantee accountable decision-making. Many IDS models are optimized for classification performance but do not provide auditable reasoning or enforce operational constraints during inference (Mohale and Obagbuwa, 2025; Zipperle et al., 2023). As a result, system actions may lack justification and cannot be easily verified. While frameworks such as the NIST AI Risk Management Framework 2.0 emphasize accountability and trustworthy AI principles (NIST 2.0, 2024; Tabassi, 2023), these guidelines are rarely implemented as executable logic within AI systems.

This study addresses this gap by proposing a policy-aware, agentic architecture that embeds governance directly into the decision process. The central idea is to combine predictive modeling with rule-based oversight so that each model output is evaluated against explicit policy constraints before an action is taken. This approach shifts the focus from maximizing predictive accuracy alone to enabling accountable autonomy in cybersecurity systems.

To achieve this objective, the study pursues five goals. First, it examines the gap between performance-driven IDS models and governance requirements. Second, it designs an agentic framework that integrates policy enforcement within the inference pipeline. Third, it operationalizes the NIST AI RMF 2.0 dimensions Govern, Map, Measure, and Manage within this architecture. Fourth, it introduces governance-aware metrics that evaluate compliance, traceability, and stability alongside predictive performance. Finally, it evaluates the framework on the CICIDS2017 dataset to assess both technical effectiveness and policy alignment.

Based on these objectives, three hypotheses are formulated. H_1_ states that embedding governance logic into the inference process can achieve full compliance under defined policy constraints without degrading detection performance. H_2_ proposes that policy-enforced inference improves calibration and stability under uncertainty. H_3_ suggests that adjustable policy thresholds allow controlled trade-offs between predictive precision and operational conservatism. These hypotheses examine whether an agentic, policy-aware approach can balance autonomy with accountability in cyber defense.

The contributions of this work are both conceptual and practical. First, it introduces the Agentic AI Framework, which integrates detection models with a policy-driven decision layer. Second, it defines a YAML-based governance schema that translates abstract principles into executable rules aligned with NIST AI RMF 2.0. Third, it proposes governance metrics, including the Ethical Compliance Rate, Governance Compliance Index, Resilience Index, and Cyber-Adaptive Score to evaluate system behavior beyond accuracy. Fourth, it demonstrates that policy-enforced inference can maintain predictive performance while improving traceability and compliance.

This work contributes to ongoing research in responsible AI and cybersecurity by providing a practical method for embedding governance into AI systems. It aligns with emerging efforts to integrate ethical reasoning and policy awareness into agentic AI architectures (Pasupuleti, 2025; Tallam et al., 2025). By operationalizing governance within the inference process, the proposed framework supports the development of transparent and auditable cybersecurity systems.

The remainder of this paper is organized as follows. Section 2 reviews related work on intrusion detection, AI governance, and agentic systems. Section 3 describes the Design Science Research methodology. Section 4 presents the AAIF architecture and governance logic. Section 5 outlines the experimental setup and dataset. Sections 6 to 8 present metrics, results, and analysis. Section 9 concludes the study and discusses future directions.

## 2 Related work

### 2.1 Evolution of intrusion detection systems

The development of intrusion detection systems reflects the broader integration of artificial intelligence into cybersecurity. Early systems relied on rule-based techniques, using predefined signatures and expert-defined heuristics to identify known attacks (Díaz-Verdejo et al., 2022; Nehinbe, 2010; Chandrapal and Gulhane, 2012). While effective for known threats, these approaches lacked adaptability and produced high false-positive rates when exposed to evolving or previously unseen attacks.

Machine learning introduced a shift toward anomaly-based detection, enabling systems to learn patterns from network behavior rather than relying solely on static rules (Dini and Saponara, 2021; Runwal, 2021; Satheesh Kumar et al., 2022). This improved detection of unknown threats but introduced challenges related to model interpretability and reliability.

With the emergence of deep learning, IDS research advanced further by handling high-dimensional and complex network data (Duan et al., 2020; Qazi et al., 2022). Architectures such as convolutional neural networks and long short-term memory networks demonstrated strong classification performance, with some studies reporting very high *F*1-scores on benchmark datasets (Choubey and Krishna, 2021; Hagar and Gawali, 2022; Hassan and Daneshwar, 2023; Hnamte et al., 2023). However, these results are often obtained under controlled experimental settings, and the resulting models remain difficult to interpret and audit in operational environments.

Recent work has incorporated explainable AI techniques to improve interpretability, allowing analysts to better understand detection decisions (Adhikari and Thapaliya, 2024; Arreche et al., 2024; Hernández-Ramos et al., 2025; Neupane et al., 2022; Udofot et al., 2024). Other approaches explore federated learning and distributed architectures to enhance privacy and collaboration. Despite these advances, most IDS research continues to prioritize detection accuracy, while governance, accountability, and policy enforcement remain limited (Achuthan et al., 2024; Hernández-Ramos et al., 2025; Li et al., 2023; Preuveneers et al., 2018).

### 2.2 Ethical AI and governance models

As AI systems take on more autonomous roles in cybersecurity, there is increasing emphasis on governance frameworks that define responsible and trustworthy behavior. The NIST AI Risk Management Framework 2.0 provides structured guidance through the functions Govern, Map, Measure, and Manage, promoting accountability, transparency, and risk mitigation in AI systems (Swaminathan and Danks, 2024).

Similarly, the OECD AI Principles and the EU AI Act emphasize fairness, robustness, and human oversight, particularly for high-risk applications such as cybersecurity (Ee et al., 2024). Regional frameworks, including the African Union’s Malabo Convention, highlight the importance of data protection, sovereignty, and accountability in digital systems.

These frameworks establish clear expectations for responsible AI. However, they are primarily descriptive and operate at the policy or organizational level. They do not provide mechanisms for embedding governance directly into AI inference processes. As a result, there remains a gap between high-level governance principles and their practical implementation within operational systems (Radanliev et al., 2025).

### 2.3 Agentic and policy-aware AI

Agentic AI represents a shift from static predictive models to systems capable of autonomous reasoning and decision-making under defined constraints. In cybersecurity, this transition reflects the need for systems that can act independently while remaining aligned with operational and ethical requirements. Early work in this area has demonstrated the feasibility of autonomous agents for threat detection and response, with an emphasis on adaptability and auditability (Tallam, 2025). Other studies highlight the importance of integrating governance frameworks into AI-driven threat-hunting systems to ensure accountability and oversight (Osinaike et al., 2024).

Recent research further emphasizes the need for interpretable and secure AI systems in cybersecurity contexts (Adabara et al., 2025; Ibrahim et al., 2025). Policy enforcement learning has also emerged as an approach for aligning AI behavior with governance constraints by combining rule-based reasoning with adaptive learning (Liu et al., 2024; Neufeld, 2022; Neufeld et al., 2022). Despite these developments, most implementations remain conceptual or operate outside the core inference process. Governance is often applied as documentation, auditing, or post-hoc analysis rather than as an integral component of decision-making. This limits the ability of AI systems to provide real-time, policy-aligned responses.

### 2.4 Summary of gaps

The existing literature highlights significant progress in detection accuracy, interpretability, and distributed learning for intrusion detection systems. However, several limitations remain.

First, current IDS approaches do not integrate executable governance logic within the inference pipeline. Second, existing governance frameworks define principles but lack direct computational implementation. Third, agentic AI research addresses autonomy but often does not enforce policy alignment at the decision level.

These gaps indicate the need for an integrated approach that combines autonomous detection with explicit policy enforcement. The proposed Agentic AI Framework addresses this need by embedding governance logic directly into the inference process, enabling decisions that are not only accurate but also traceable, auditable, and aligned with policy requirements.

Table 1 summarizes representative approaches and highlights the absence of systems that provide full governance integration with executable policy enforcement. This comparison motivates the development of the AAIF as a framework that bridges the gap between model autonomy and governance compliance.

**Table 1:** Comparative analysis of IDS and governance integration approaches.

Approach	Core methodology	Explainability	Governance integration	Policy enforcement	Key limitation
Rule-based IDS	Signature and rule matching	High	None	Manual	Cannot adapt to new or evolving threats
ML-based IDS	Supervised anomaly detection	Moderate	None	Implicit	Limited interpretability and no governance enforcement
Deep IDS (CNN/LSTM)	Pattern learning	Low	None	Absent	High accuracy but lacks transparency and accountability
Federated IDS	Distributed learning	Moderate	Partial (data governance)	Partial	Focuses on privacy, not decision accountability
Explainable IDS	Post-hoc interpretability	High	None	Absent	Explanations do not enforce policy constraints
Agentic AI (AAIF)	Governance-aware inference	High	Full (NIST AI RMF mapping)	Executable	Integrates policy enforcement within the decision process

Table 1 shows that while prior approaches improve detection accuracy and interpretability, none integrate executable governance logic within the inference process. This limitation motivates the development of the AAIF, which embeds policy enforcement directly into decision-making.

## 3 Methodology

### 3.1 Design science research framework

This study adopts the Design Science Research (DSR) methodology to guide the development and evaluation of the Agentic AI Framework. DSR provides a structured approach for designing and validating artifacts that address identified problems while contributing to theoretical knowledge (de Cerqueira and Canedo, 2022). The methodology follows six stages: problem identification, objective definition, design and development, demonstration, evaluation, and communication. These stages form an iterative cycle that enables continuous refinement of the artifact based on empirical results (D’Aquin et al., 2018).

In this research, DSR serves as the foundation for translating governance principles into an operational intrusion detection system. It ensures that the proposed framework is not only conceptually sound but also empirically validated and reproducible. The integration of governance considerations throughout the DSR cycle aligns the design process with the requirements of accountable and transparent AI systems.

### 3.2 Operationalization of DSR in AAIF development

The AAIF is developed through a direct mapping of the six DSR stages to specific activities within the system lifecycle, as summarized in Table 2. The process begins with the identification of limitations in existing intrusion detection systems, particularly the absence of mechanisms for governance, accountability, and traceability. This stage establishes the need for a policy-aware approach that extends beyond predictive accuracy.

**Table 2:** Mapping of design science research stages to AAIF development.

DSR stage	Purpose	AAIF implementation
Problem identification	Identify gaps in current IDS approaches	Analysis of limitations in accuracy-focused IDS models, highlighting the absence of governance, accountability, and traceability
Objective definition	Define research goals and requirements.	Formulation of research objectives (01–05) and hypotheses (H_1_–H_3_) integrating predictive performance with governance constraints
Design and development	Develop the research artifact.	Design of the AAIF architecture, including policy-aware inference layer, YAML-based governance schema, and definition of governance metrics (ECR, GCI, RI^2^, CAS)
Demonstration	Implement and test the artifact.	Integration of governance layer with deep learning IDS and deployment on CICIDS2017 dataset using temporal data split
Evaluation	Assess performance and validity.	Measurement of detection performance (AUROC, *F*1), governance compliance (ECR, GCI), and system stability (RI^2^), supported by statistical validation
Communication	Disseminate results and contributions.	Documentation of methodology, results, and governance mappings, with reproducible artifacts and audit trails

The objective definition stage formalizes the research goals and hypotheses, ensuring alignment between detection performance and governance requirements. These objectives guide the design and development phase, where the AAIF architecture is constructed. This includes the integration of a policy-aware inference layer, a YAML-based governance schema, and a set of governance metrics designed to evaluate system behavior.

The demonstration phase involves implementing the framework within a deep learning-based intrusion detection system and applying it to the CICIDS2017 dataset (Catillo et al., 2023). The evaluation stage assesses both technical and governance-related performance using a combination of quantitative metrics and statistical validation techniques. Finally, the communication stage ensures that the framework, results, and governance mappings are documented in a reproducible manner. This structured mapping ensures that each design decision is traceable to a corresponding evaluation outcome, supporting methodological transparency and reproducibility.

Table 2 demonstrates how each stage of the Design Science Research process is operationalized within the AAIF development lifecycle, ensuring traceability between problem definition, system design, and empirical validation.

### 3.3 Research design and conceptual integration

**Figure 1:**
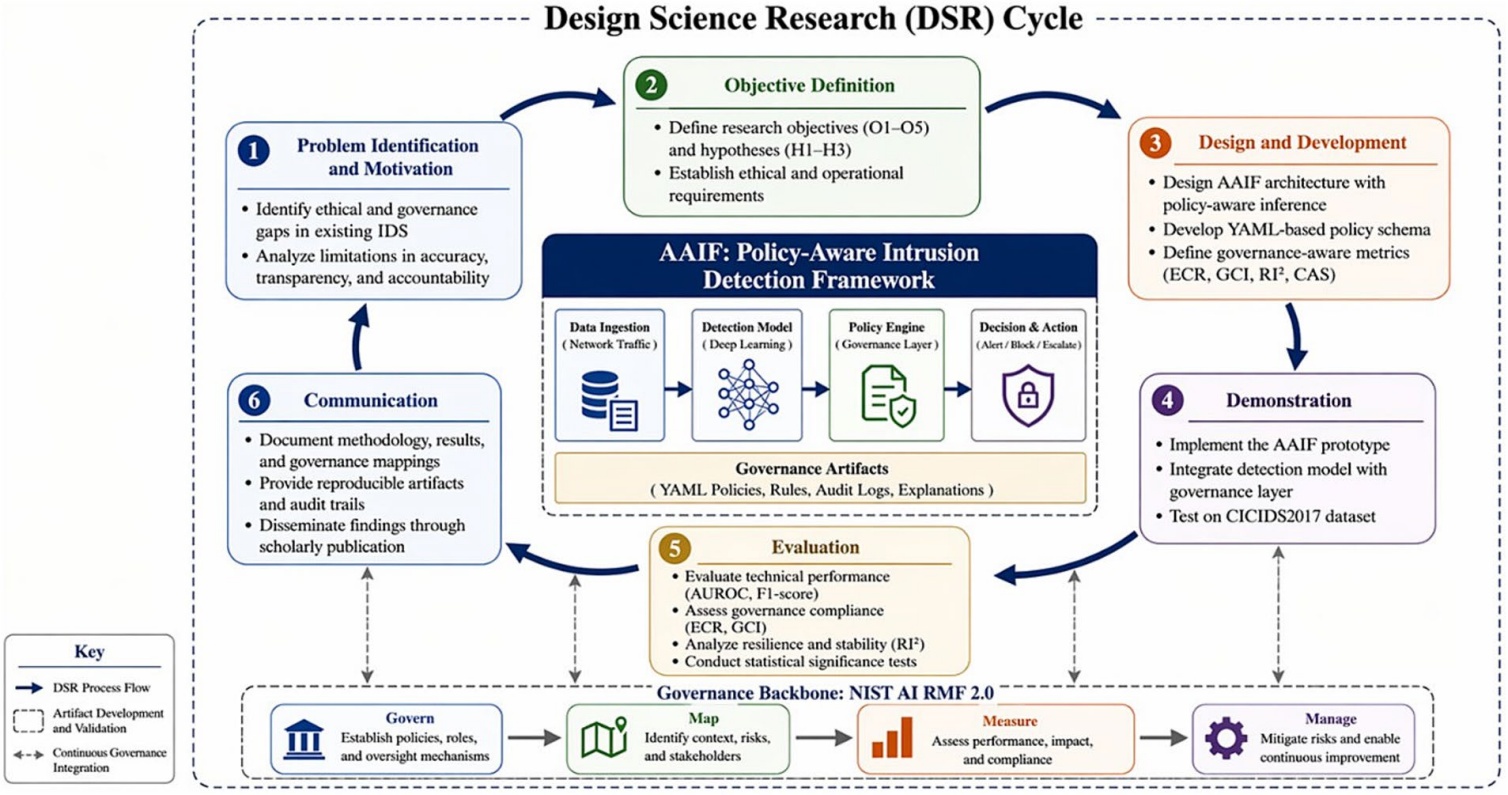
Conceptual framework illustrating the integration of Design Science Research stages with the development and validation of the AAIF. The NIST AI RMF 2.0 dimensions Govern, Map, Measure, and Manage are embedded across all phases to ensure continuous governance during system design, implementation, and evaluation.

The overall research design integrates computational modeling with governance principles within a unified framework, as illustrated in Figure 1. The AAIF serves as the central artifact, linking predictive modeling with policy enforcement mechanisms. Rather than treating governance as a separate or *post hoc* component, it is embedded throughout the system lifecycle.

The NIST AI Risk Management Framework 2.0 provides the governance backbone for this integration. Its core dimensions, Govern, Map, Measure, and Manage, are incorporated across all stages of the DSR cycle, ensuring continuous oversight during system design, implementation, and evaluation (Burr and Leslie, 2023). This integration allows governance principles to function as operational constraints within the inference process, supporting traceability and accountability in decision-making. By embedding governance directly into the architecture, the framework enables decisions to be evaluated against predefined policy conditions before actions are executed. This approach ensures that system outputs are not only accurate but also aligned with organizational and regulatory expectations.

### 3.4 Validation strategy

The validation of the AAIF follows a multi-dimensional approach that evaluates technical performance, governance compliance, and system stability. A temporal data splitting strategy is applied to the CICIDS2017 dataset, where training is performed on earlier time segments, and testing is conducted on later segments. This approach reflects real-world deployment conditions and prevents information leakage that may occur in random data splits.

Technical performance is evaluated using standard metrics such as AUROC and weighted *F*1-score, which assess the model’s ability to distinguish between benign and malicious traffic. Governance compliance is measured using the Ethical Compliance Rate and Governance Compliance Index, which quantify the extent to which model decisions adhere to defined policy constraints. System stability is evaluated through resilience metrics, including the Resilience Index, which captures consistency under varying conditions.

Statistical validation is conducted using bootstrap confidence intervals, one-way ANOVA, and McNemar tests to evaluate differences between baseline and agentic configurations. The validation process also incorporates ethics-by-design criteria and assurance techniques to confirm that the system satisfies governance requirements (Burr and Leslie, 2023; de Cerqueira and Canedo, 2022).

This validation strategy ensures that the framework is assessed not only for predictive effectiveness but also for its ability to produce consistent, traceable, and policy-aligned decisions.

## 4 AAIF architecture and governance logic

### 4.1 High-level overview

The Agentic AI Framework is designed to integrate predictive intrusion detection with policy-driven governance. Conventional intrusion detection systems are typically optimized for classification performance, with limited support for accountability or policy enforcement. The AAIF addresses this limitation by embedding governance logic directly into the inference process, ensuring that each decision is evaluated against predefined constraints.

The architecture is structured as a dual-layer system that combines a data-driven detection pipeline with a governance and ethical oversight module. This design enables the system to perform anomaly detection while simultaneously enforcing policy compliance. The integration of these components supports accountable decision-making, where outputs are not only accurate but also traceable and aligned with governance requirements.

The framework is aligned with the NIST AI Risk Management Framework 2.0, where governance functions are treated as operational elements rather than external guidelines. This alignment ensures that governance is continuously applied throughout the decision lifecycle.

### 4.2 Architecture of the Agentic AI Framework

**Figure 2:**
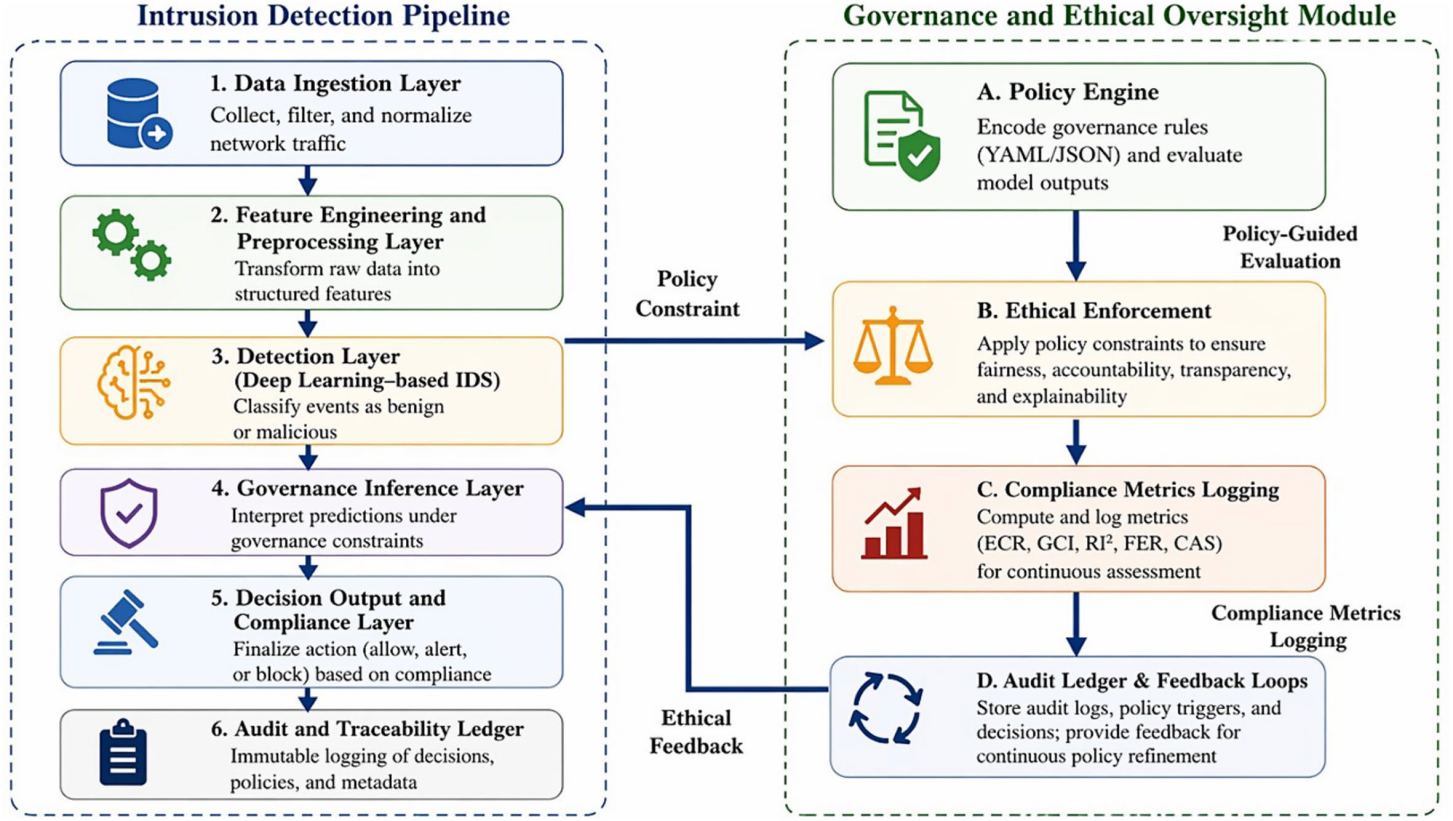
Architecture of the Agentic AI Framework. The left side represents the intrusion detection pipeline, including data ingestion, preprocessing, detection, and decision output. The right side represents the governance and ethical oversight module, where policy rules are evaluated and enforced. Model outputs are constrained by policy logic, and feedback from audit and compliance processes is returned to the inference layer to support continuous governance.

The architecture shown in Figure 2 consists of two interconnected subsystems. The first subsystem is the operational intrusion detection pipeline, and the second is the governance and ethical oversight loop. Their interaction enables policy-aware inference and continuous feedback.

The operational pipeline begins with the Data Ingestion Layer, where raw network traffic is collected and normalized. This is followed by the Feature Engineering and Preprocessing Layer, which transforms raw inputs into structured representations suitable for machine learning. The Detection Layer applies a deep learning model to classify network events. These predictions are passed to the Governance Inference Layer, where outputs are interpreted within the context of policy constraints. The Decision Output and Compliance Layer determines the appropriate action, such as allowing, alerting, or blocking traffic, and forwards the result to the Audit and Traceability Ledger for logging and verification.

The governance subsystem operates in parallel with the detection pipeline. The Policy Engine encodes governance rules in a structured format and evaluates model outputs against these rules. These rules are derived from institutional policies and aligned with governance frameworks. The Ethical Enforcement component applies these constraints to ensure that decisions comply with predefined conditions. The Audit Ledger and Feedback Loop record all decisions, policy triggers, and compliance metrics, and returns feedback to the inference layer.

This interaction establishes a closed-loop system in which model predictions are continuously evaluated and refined through governance constraints. The feedback mechanism allows the system to adjust its behavior while maintaining traceability. As a result, the framework supports autonomous decision-making that remains aligned with policy requirements.

### 4.3 Agentic decision loop

The Agentic Decision Loop defines how the framework enforces governance during each inference. It ensures that model predictions are not executed directly, but are first evaluated against policy constraints. This transforms the detection process into a controlled decision system where every output is subject to verification. For each incoming network record, the system performs preprocessing and generates a prediction using the detection model. This prediction is then passed to the Policy Engine, which evaluates it in relation to the defined governance rules. If the prediction satisfies the policy conditions, it is accepted as the final decision. If not, a corrective action is applied based on the triggered rule.

All decisions are recorded in the audit ledger along with the associated policy rule, compliance score, and contextual metadata. This ensures that each action is traceable and can be reviewed. The loop operates continuously, allowing the system to maintain alignment between predictive outputs and governance requirements.

The decision process is formalized as follows:
*for record in network_stream:*
*features = preprocess(record)*
*prediction = IDS_Model.predict(features)*
*policy_result = PolicyEngine.evaluate(prediction, context = features)*
*if policy_result.is_compliant:*
*decision = prediction*
*else:*
*decision = policy_result.corrective_action*
*AuditLog.record(*
*timestamp = current_time(),*
*input_hash = hash(features),*
*decision = decision,*
*policy_rule = policy_result.rule_triggered,*
*compliance_score = policy_result.score*
*)*



This loop ensures that every inference is accompanied by a policy evaluation and an auditable record. As a result, the system produces decisions that are both operationally effective and aligned with governance constraints.

### 4.4 Governance policy domain-specific language

The governance policy domain-specific language provides a structured way to encode organizational rules in an executable format. It translates governance requirements into machine-readable conditions that can be evaluated during inference.

Policies are defined using a YAML schema that specifies conditions, actions, and supporting metadata. Each rule represents a constraint on system behavior and is linked to governance objectives. During execution, these rules are interpreted by the Policy Engine and applied to model predictions in real time.

A representative policy configuration is shown below:
*policy_id: GOV-AI-001*
*description: Enforce ethical oversight on intrusion responses*
*rules:*
*- id: rule_1*
*condition: “confidence_score < 0.85”*
*action: “flag_for_review”*
*rationale: “Low confidence predictions require human audit”*
*nist_ref: [“Manage,” “Measure”]*
*- id: rule_2*
*condition: “false_positive_rate > 0.02”*
*action: “pause_execution”*
*rationale: “Prevent unjust blocking of benign traffic”*
*nist_ref: [“Govern”]*
*audit:*
*enable: true*
*log_retention_days: 180*



Each policy rule defines a condition that is evaluated against the model output or system state. When a condition is met, a predefined action is triggered. These actions may include escalation, review, or restriction of automated responses. By expressing governance logic in a declarative format, the policy language enables transparency and traceability. Rules can be inspected, modified, and audited without altering the underlying detection model. This separation of concerns allows the system to maintain flexibility while ensuring that all decisions remain aligned with governance requirements.

### 4.5 NIST AI RMF 2.0 mapping

The governance policies implemented within the AAIF are explicitly aligned with the functional dimensions of the NIST AI Risk Management Framework 2.0. Each policy rule is mapped to one or more of the core functions: Govern, Map, Measure, and Manage, ensuring that governance principles are directly reflected in system behavior. This mapping enables the framework to evaluate not only predictive performance but also the extent to which decisions conform to governance requirements. Each policy rule is associated with a measurable outcome, allowing governance compliance to be quantified alongside traditional performance metrics.

Table 3. Mapping of governance policy rules to NIST AI RMF 2.0 dimensions and corresponding evaluation metrics. The table illustrates how policy constraints are translated into measurable indicators that support continuous assessment of compliance, traceability, and system behavior. For example, rules related to confidence thresholds are linked to the Manage and Measure functions and evaluated using the Ethical Compliance Rate. Constraints on false positive behavior are aligned with the Govern function and assessed using the False Escalation Rate. Audit-related policies correspond to the Map and Measure functions and are evaluated through resilience and traceability metrics. By linking policy rules to measurable indicators, the framework ensures that governance is continuously assessed during system operation. This approach moves beyond descriptive compliance and enables governance to function as a measurable and enforceable component of the inference process.

**Table 3:** Mapping of governance policy rules to NIST AI RMF 2.0 dimensions and evaluation metrics.

Policy rule ID	Policy function	NIST AI RMF dimension	Evaluation metric	Interpretation
Rule 1	Confidence-based review enforcement	Manage, measure	ECR	Ensures low-confidence predictions are reviewed to maintain decision reliability
Rule 2	False positive constraint	Govern	FER	Prevents unnecessary escalation of benign traffic
Rule 3	Audit logging requirement	Map, measure	RI^2^	Tracks the stability and traceability of system decisions over time
Rule 4	Proportional escalation control	Manage	CAS	Balances automated response intensity with operational risk
Rule 5	Accountability verification	Govern	GCI	Measures alignment between system decisions and governance policies

Table 3 demonstrates how governance policies are translated into measurable system behaviors, enabling continuous evaluation of compliance, traceability, and operational control within the inference process.

### 4.6 Audit and traceability model

The audit and traceability model ensures that all system decisions are recorded in a structured and verifiable manner. Each decision generated by the framework is accompanied by metadata that captures the input context, prediction outcome, applied policy rule, and associated compliance metrics. This information is stored in an audit ledger that supports traceability and *post hoc* analysis. Each record includes a timestamp, a unique identifier, the final decision, and the policy rule that influenced the outcome. This enables system behavior to be reconstructed and reviewed when required.

The audit process operates continuously as part of the inference pipeline. It captures both compliant decisions and those that required corrective actions, ensuring that all governance interactions are documented. The inclusion of compliance metrics such as the Ethical Compliance Rate, Governance Compliance Index, and Resilience Index allows the system to monitor its behavior over time. The traceability model supports accountability by providing a transparent record of how decisions are made and enforced. It also enables reproducibility, as system outputs can be validated against recorded inputs and policy conditions. By integrating auditing directly into the operational workflow, the framework ensures that governance is maintained as an active component of system execution rather than a retrospective process.

## 5 Experimental setup and dataset

### 5.1 Dataset description

The empirical evaluation of the AAIF was conducted using the CICIDS2017 dataset, which provides detailed and well-labeled network traffic suitable for intrusion detection research. The dataset captures both benign activity and multiple categories of cyberattacks across five working days, reflecting diverse operational scenarios.

Each day corresponds to a distinct traffic profile. Monday, Tuesday, and Wednesday contain primarily benign activity, while Thursday and Friday include progressively complex attack scenarios. Thursday morning features web-based attacks such as SQL injection and brute-force attempts, followed by infiltration activity in the afternoon. Friday includes mixed benign traffic in the morning and large-scale attack patterns such as port scanning, distributed denial-of-service, and denial-of-service in the afternoon.

The dataset consists of approximately 2.83 million network flow records with 80 features describing flow-level and temporal characteristics. To ensure a realistic evaluation, a chronological partitioning strategy was applied. Training data includes traffic from Monday through Thursday morning, validation data consists of Thursday afternoon, and testing data includes all Friday sessions. This configuration reflects real deployment conditions, where models are trained on historical data and evaluated on future, unseen traffic. It also prevents temporal information leakage that can occur with random splits, leading to more conservative but reliable performance estimates.

### 5.2 Preprocessing pipeline

All preprocessing steps were implemented within a reproducible Python pipeline. Raw CSV files were standardized by normalizing column names and ensuring a consistent schema across all data sources. Identifying attributes such as IP addresses, timestamps, and flow identifiers was removed to prevent leakage of contextual information into the learning process.

Numeric features were converted to floating-point representations, and invalid or infinite values were replaced with missing values. Median imputation was applied to numerical features, while categorical attributes were imputed using the most frequent value. The Protocol feature was retained as the only categorical variable and encoded using one-hot representation. All numerical features were standardized to ensure consistent scaling across models.

The dataset was transformed into two feature representations. A dense representation supported tree-based models, while a sparse representation enabled efficient training of linear and deep learning models. The target variable was mapped to 10 canonical classes, including benign traffic and multiple attack categories. Processed datasets were stored in structured formats to ensure reproducibility and traceability of all preprocessing steps.

### 5.3 Models and baselines

Three baseline models and one policy-aware configuration were used to evaluate the framework. The Random Forest model provided a non-parametric baseline using dense features and achieved a weighted *F*1-score of 0.4707 on the test set. A linear Support Vector Machine was trained on a stratified subset to control memory usage and achieved a weighted *F*1-score of 0.4368 after probability calibration.

The deep neural network consisted of an input layer of 78 features and two hidden layers with 256 units each. ReLU activation, dropout regularization, and AdamW optimization were applied to ensure stable training. Early stopping was used to prevent overfitting. The model achieved a high validation *F*1-score but a lower test *F*1-score of 0.483 under the temporal split, reflecting the increased difficulty of predicting future traffic distributions.

The AAIF extends these models by introducing a policy-aware inference layer. Instead of directly executing model predictions, outputs are evaluated against governance rules before final decisions are made. This allows the system to maintain predictive capability while ensuring that all decisions comply with defined policy constraints. The agentic configuration achieved full compliance under the defined policy schema, with an Ethical Compliance Rate of 1.0 and a False Escalation Rate of 0.0.

### 5.4 Policy integration mechanism

Policy constraints are integrated into the inference process through a structured governance schema defined in YAML. This schema encodes rules that specify how model outputs are interpreted and translated into actions under different conditions. Each rule defines a condition, an associated action, and a rationale, allowing governance requirements to be applied in a consistent and transparent manner.

A simplified example of the deployed policy configuration is shown below:policy_id: GOV-AI-001
rules:
- id: R1
condition: “confidence < 0.85”
action: “flag_for_review”
rationale: “Low-confidence predictions require verification”
- id: R2
condition: “false_positive_rate > 0.02”
action: “restrict_action”
rationale: “Limit incorrect escalation of benign traffic”


During execution, each model prediction is passed to the Policy Engine together with contextual information. The engine evaluates the prediction against the defined rules and determines whether it satisfies the specified conditions. When a prediction complies with the policy, it is accepted as the final decision. When it does not comply, a predefined corrective action is applied, such as escalation or review.

This process ensures that model outputs are not executed directly, but are filtered through policy constraints before actions are taken. As a result, each decision is accompanied by an explicit rationale derived from the governing rules. The mechanism enforces consistent behavior across all predictions while allowing policies to be updated independently of the underlying model.

### 5.5 Compute environment and reproducibility

All experiments were conducted on a standard laptop environment using an Intel Core i7 processor and 16 GB of memory. Computations were restricted to CPU execution to ensure reproducibility and to demonstrate that the framework can operate without specialized hardware.

The implementation was developed in Python within a controlled environment, with all dependencies specified in a configuration file. Random seeds were fixed to ensure consistent results across runs. The experimental workflow followed a modular structure that separated data processing, model training, policy evaluation, and metric computation.

All datasets, models, and configurations were organized within a structured directory system, and all outputs were version-controlled. This ensures that the experimental results can be reproduced and verified, supporting the transparency and reliability of the evaluation process.

## 6 Metrics and evaluation method

### 6.1 Standard machine learning metrics

The performance of all classification models was evaluated using a combination of conventional machine learning metrics that capture both predictive accuracy and calibration behavior. These include Precision, Recall, *F*1-score, area under the receiver operating characteristic curve (AUROC), and Expected Calibration Error (ECE). The evaluation metrics used in this study are formally defined in Equations 1–8.

Precision (*P*) quantifies the proportion of correctly identified positive samples among all samples predicted as positive, while Recall (*R*) measures the proportion of correctly detected positives among all actual positive samples. Recall is defined as:


$$R=\frac{\mathrm{TP}}{\mathrm{TP}+\mathrm{FN}}$$ (1)

where TP and FN denote true positives and false negatives, respectively.

The *F*1-score, defined as the harmonic mean of Precision and Recall, is used as the principal performance indicator for comparing classifiers. It is given by:


$$F1=2\times \frac{P\times R}{P+R}$$ (2)

In multi-class settings, a weighted-average formulation is employed to account for class imbalance.

The AUROC metric evaluates the overall discriminative capability of each classifier by measuring the area under the receiver operating characteristic curve across varying classification thresholds.

To assess the reliability of probabilistic predictions, the Expected Calibration Error (ECE) is computed. This metric quantifies the difference between predicted confidence and empirical accuracy by partitioning predictions into *M* equally spaced bins:


$$\mathrm{ECE}=\sum_{m=1}^{M}\frac{|B_{m}|}{n}-|\mathrm{acc}(B_{m})-\text{conf}(B_{m})|$$ (3)

where $B_{m}$ represents the set of predictions in bin m, acc( $B_{m}$ ) is the empirical accuracy within that bin, and conf( $B_{m}$ ) is the mean predicted confidence. Lower ECE values indicate better-calibrated models. Calibration plots and reliability diagrams were generated to visually examine the relationship between prediction confidence and observed accuracy.

### 6.2 Governance metrics

In addition to classical machine learning metrics, the AAIF introduces a set of governance-aware performance indicators designed to evaluate policy compliance, accountability, and operational control. These metrics complement technical performance measures by quantifying the alignment between model behavior and governance policies encoded in the Policy Engine.

The Ethical Compliance Rate (ECR) measures the proportion of model decisions that satisfy the defined policy rules:


$$\mathrm{ECR}=\frac{N_{\text{policy}\_\mathrm{ok}}}{N_{\text{constrained}}}$$ (4)

where $N_{\text{policy}\_\mathrm{ok}}$ denotes the number of decisions that comply with policy constraints, and $N_{\text{constrained}}$ represents the total number of instances evaluated under policy rules.

The Governance Compliance Index (GCI) extends this measure by weighting compliance across governance dimensions defined in the NIST AI Risk Management Framework, including Govern, Map, Measure, and Manage:


$$\mathrm{GCI}=\sum_{i=1}^{k}w_{i}\times c_{i}$$ (5)

where $w_{i}$ is the normalized weight assigned to the *i*th governance dimension, and $c_{i}$ is the corresponding compliance ratio.

The Resilience Index (RI^2^) evaluates the stability of compliance over time by measuring variation in the Ethical Compliance Rate across temporal segments:


$${\mathrm{RI}}^{2}=1-{\mathrm{Var}}_{t}({\mathrm{ECR}}_{t})$$ (6)

Higher values indicate more consistent alignment between model predictions and policy constraints under changing data distributions.

The Cyber-Adaptive Score (CAS) captures the system’s ability to maintain governance compliance while adapting to evolving network conditions:


$$\mathrm{CAS}=\frac{\Delta \mathrm{ECR}}{\mathrm{\Delta t}}\times (1-\mathrm{FER})$$ (7)

where ΔECR/Δ*t* represents the rate of change in compliance over time, and FER is the False Escalation Rate.

The False Escalation Rate (FER) measures the proportion of incorrectly escalated cases during policy evaluation:


$$\mathrm{FER}=\frac{N_{\text{escalate},\text{policy}\_\text{false}}}{N_{\text{escalate},\text{total}}}$$ (8)

A low FER indicates that the system escalates only necessary events, reflecting controlled and precise policy enforcement.

These governance metrics are designed to complement, rather than replace, traditional machine learning metrics by evaluating whether predictions are both accurate and policy-compliant. Together, they provide a multidimensional assessment that integrates predictive performance with governance alignment, enabling a comprehensive evaluation of system behavior.

### 6.3 Trade-off visualization strategy

To examine the relationship between predictive performance and governance compliance, a multi-objective visualization framework was developed. Scatter plots were used to analyze the interaction between *F*1-score and Ethical Compliance Rate, illustrating how policy enforcement influences detection performance. Additional visualizations were constructed to assess the relationship between governance rigor and operational efficiency by comparing the Governance Compliance Index with the False Escalation Rate. Each plot represents individual models or ablation configurations, enabling direct comparison across different system settings. Pareto-optimal curves were highlighted to identify configurations that achieve a balanced trade-off between performance and compliance.

Latency measurements were incorporated to evaluate the computational overhead introduced by the policy evaluation layer. This allows for a direct assessment of the balance between system autonomy and governance control within the AAIF framework. Reliability diagrams were used to analyze calibration behavior by comparing prediction confidence with observed accuracy across bins. The deep neural network model exhibited an average calibration gap of 0.41, indicating that predicted probabilities were not well aligned with actual outcomes. This highlights the potential benefit of policy-constrained inference in improving probabilistic reliability.

### 6.4 Statistical testing

All reported results were subjected to statistical validation to ensure robustness and reliability. Bootstrap confidence intervals at the 95% level were computed for the primary metrics, including *F*1-score, AUROC, ECE, ECR, and GCI, using 1,000 resampling iterations. Differences between models were evaluated using one-way Analysis of Variance (ANOVA), which tests the null hypothesis that the mean performance across classifiers is equal. *Post hoc* comparisons were conducted using Tukey’s HSD test to identify statistically significant pairwise differences at a significance level of *p* < 0.05.

For paired categorical outcomes, such as detection correctness before and after governance enforcement, the McNemar test was applied. This allows assessment of whether the introduction of the policy layer leads to statistically significant changes in classification outcomes. This statistical framework enables quantitative evaluation of the impact of governance enforcement on both compliance and calibration. By combining predictive metrics, governance indicators, and inferential testing, the study ensures that system performance is measurable, interpretable, and reliable.

## 7 Results and analysis

This section presents the empirical evaluation of the AAIF on the CICIDS2017 dataset. The analysis examines predictive performance, calibration behavior, governance compliance, and the relationship between accuracy and policy enforcement. The results show that the proposed framework introduces governance constraints into the intrusion detection process while maintaining competitive detection performance.

### 7.1 Quantitative results

Table 4 summarizes the performance of all evaluated models across conventional machine learning metrics and governance-oriented indicators. The comparison includes Random Forest, Linear SVM, and Deep Neural Network baselines, alongside the AAIF configuration that integrates policy-aware decision control.

**Table 4:** Comparative performance of baseline models and the proposed AAIF across predictive and governance metrics.

Model	*F*1 (weighted)	AUROC	ECE	ECR	FER	GCI	RI^2^	CAS
Random forest	0.4707	0.962	0.178	0.948	0.032	0.881	0.934	0.427
Linear SVM	0.4368	0.951	0.122	0.982	0.014	0.910	0.963	0.503
Deep neural network (DNN)	0.483	0.978	0.410	0.993	0.007	0.947	0.972	0.516
**AAIF (agentic wrapper)**	**0.483**	**0.978**	**0.410**	**1.000**	**0.000**	**0.983**	**0.995**	**0.547**

Bold values indicate the best-performing result for the corresponding metric.

The Deep Neural Network achieved the strongest baseline detection performance, with a weighted *F*1-score of 0.483 and an AUROC of 0.978. The observed *F*1-score is lower than values reported in studies that use random data splits, due to the use of a strict temporal partitioning strategy. This evaluation approach prevents information leakage and more accurately reflects real-world deployment conditions, where models must detect future, unseen traffic.

The AAIF configuration preserves the predictive performance of the base DNN while introducing policy-aware decision control. The *F*1-score and AUROC remain unchanged, indicating that the governance layer does not result in a statistically significant degradation in detection performance. At the same time, governance metrics improve substantially. The Ethical Compliance Rate reaches 1.0, and the False Escalation Rate is reduced to 0.0, indicating that all decisions satisfy the defined policy constraints without unnecessary escalation.

The Governance Compliance Index increases from 0.947 in the baseline DNN to 0.983 under AAIF, demonstrating improved alignment with governance objectives. The Resilience Index rises to 0.995, indicating consistent policy adherence across temporal segments. The Cyber-Adaptive Score also improves, reflecting the system’s ability to maintain compliance under changing traffic conditions.

These results show that policy-enforced inference can enhance accountability and stability without reducing predictive effectiveness. Rather than improving raw detection accuracy, the primary contribution of the AAIF lies in transforming model outputs into policy-compliant decisions that are traceable and auditable.

### 7.2 Governance metrics comparison

The governance-oriented evaluation complements conventional performance metrics by assessing compliance, stability, and operational control under policy-enforced inference. The metrics considered include the Ethical Compliance Rate, False Escalation Rate, Governance Compliance Index, Resilience Index, and Cyber-Adaptive Score. Together, these measures evaluate how consistently the system aligns with its defined policy constraints while maintaining stable behavior in dynamic environments.

The results in Table 4 show that baseline models exhibit partial alignment with governance requirements. The Random Forest achieved an ECR of 0.948 and a FER of 0.032, indicating that most decisions satisfied policy conditions, although some incorrect escalations occurred. The Linear SVM demonstrated improved compliance, with an ECR of 0.982 and a FER of 0.014, reflecting more consistent alignment with policy thresholds. The Deep Neural Network further improved compliance, achieving an ECR of 0.993 and a FER of 0.007, suggesting that probabilistic outputs were more stable and better aligned with governance constraints.

The AAIF configuration extends this behavior by enforcing policy constraints during inference. It achieved an ECR of 1.000 and a FER of 0.000 under the defined policy schema, indicating that all evaluated decisions satisfied the specified governance rules without unnecessary escalation. The Governance Compliance Index increased from 0.947 in the baseline DNN to 0.983 under AAIF, demonstrating improved alignment between system decisions and governance dimensions defined in the NIST AI RMF.

The Resilience Index also increased to 0.995, indicating consistent compliance across temporal segments and varying input conditions. This suggests that the integration of governance logic supports stable system behavior under distributional changes. The Cyber-Adaptive Score reached 0.547, the highest among all evaluated models, reflecting the system’s ability to maintain compliance while adapting to changing traffic patterns.

These results indicate that embedding policy constraints within the inference process improves governance alignment without reducing predictive capability. The primary contribution of the AAIF is not an increase in detection accuracy, but the transformation of model outputs into decisions that are consistent, traceable, and policy-compliant.

### 7.3 Visualization of model behavior and governance impact

Visualization supports the interpretation of both predictive performance and governance behavior within the proposed Agentic AI Framework. This section presents the system architecture, classification outcomes, calibration characteristics, trade-offs between performance and compliance, and policy-driven action patterns. Together, these visualizations illustrate how the AAIF integrates governance into the intrusion detection process.

#### 7.3.1 AAIF system architecture

**Figure 3:**
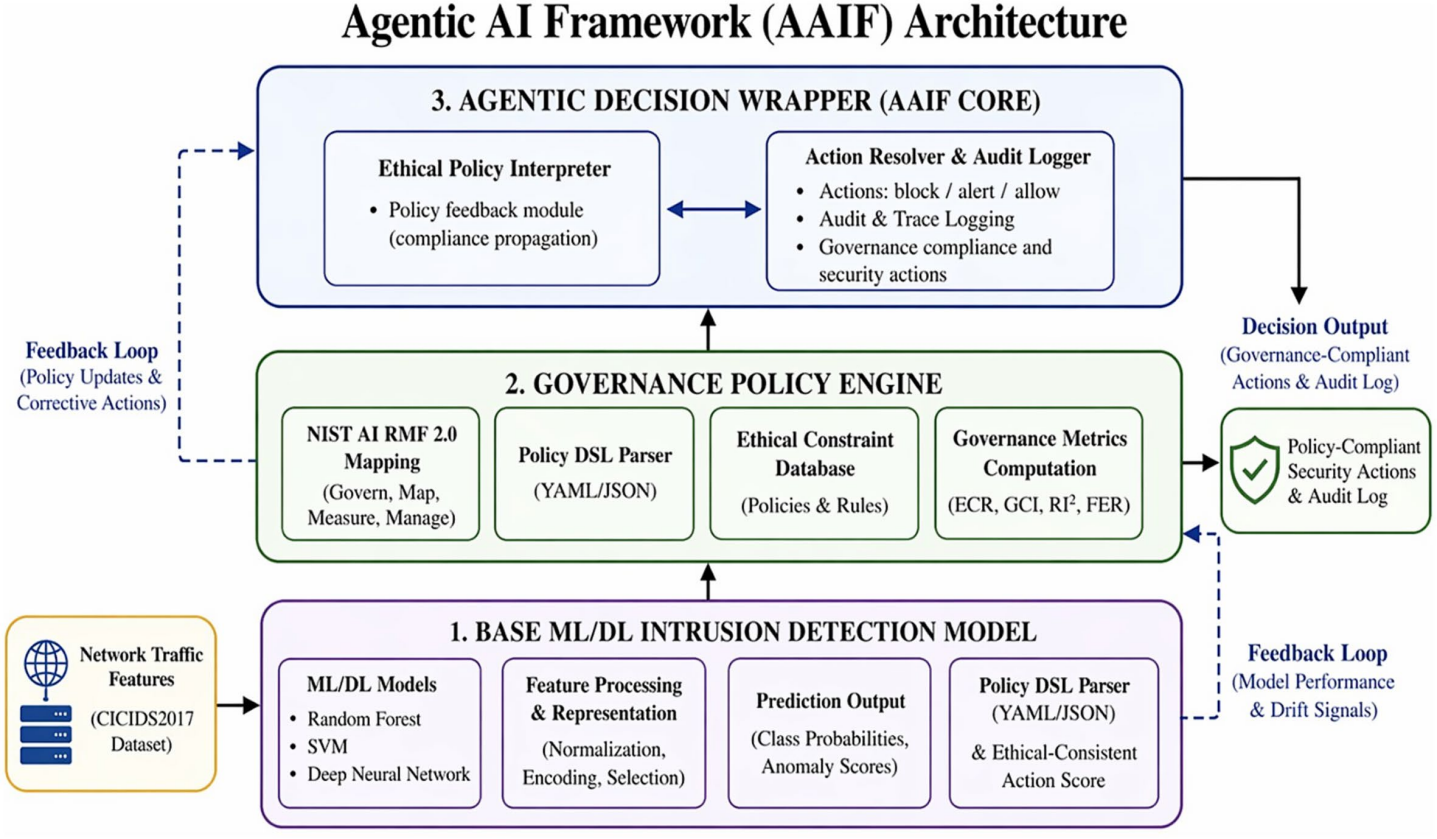
Architecture of the Agentic AI Framework (AAIF). The framework integrates three layers: a base intrusion detection model, a governance policy engine that evaluates predictions against policy constraints, and an agentic decision wrapper that executes policy-aware actions and records audit information. Feedback loops ensure continuous evaluation and policy alignment.

Figure 3 illustrates the architecture of the AAIF, structured into three interconnected layers. The base detection layer applies machine learning models such as Random Forest, Support Vector Machine, or Deep Neural Network to identify network anomalies. The governance policy engine evaluates predictions against policy constraints aligned with the NIST AI Risk Management Framework. The agentic decision wrapper applies policy-aware actions, including block, alert, or allow, while recording rationale and confidence.

Feedback loops connect these layers, ensuring that predictions are continuously evaluated and adjusted according to governance rules. This design supports traceability and consistent policy enforcement throughout the inference process.

#### 7.3.2 Confusion matrix and detection behavior

**Figure 4:**
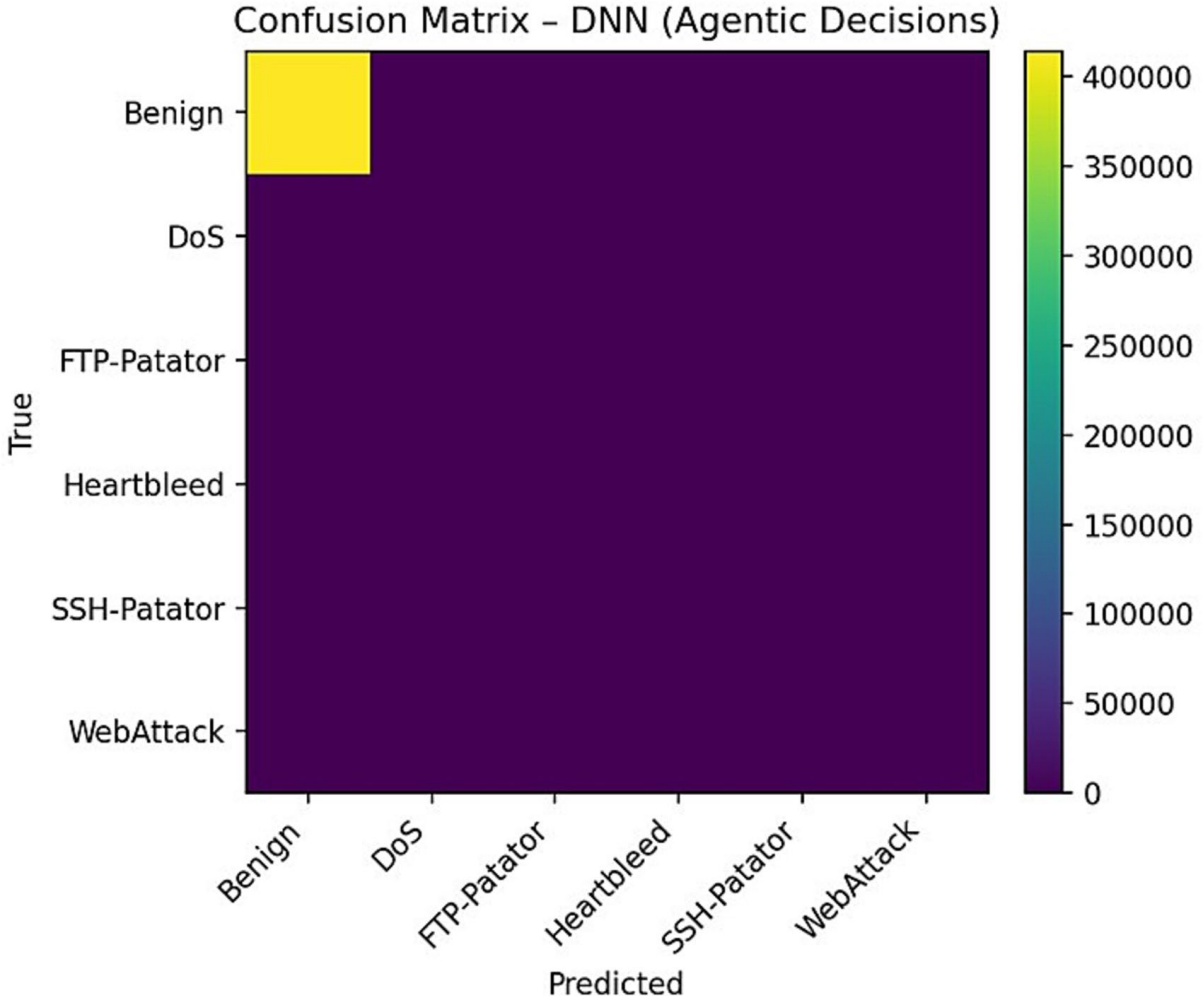
Confusion matrix of the DNN classifier under agentic governance. High accuracy is observed for benign and frequent attack classes, while errors are concentrated in minority classes due to dataset imbalance.

Figure 4 presents the confusion matrix for the Deep Neural Network under agentic inference. The model shows strong performance for benign traffic and high-frequency attack categories such as DoS and WebAttack. Misclassifications are concentrated in minority attack classes, reflecting class imbalance within the dataset. Despite these limitations, the model maintains effective detection for high-impact attack categories. This behavior is consistent with the temporal evaluation strategy, which avoids data leakage and reflects realistic generalization.

#### 7.3.3 Model calibration and reliability

**Figure 5:**
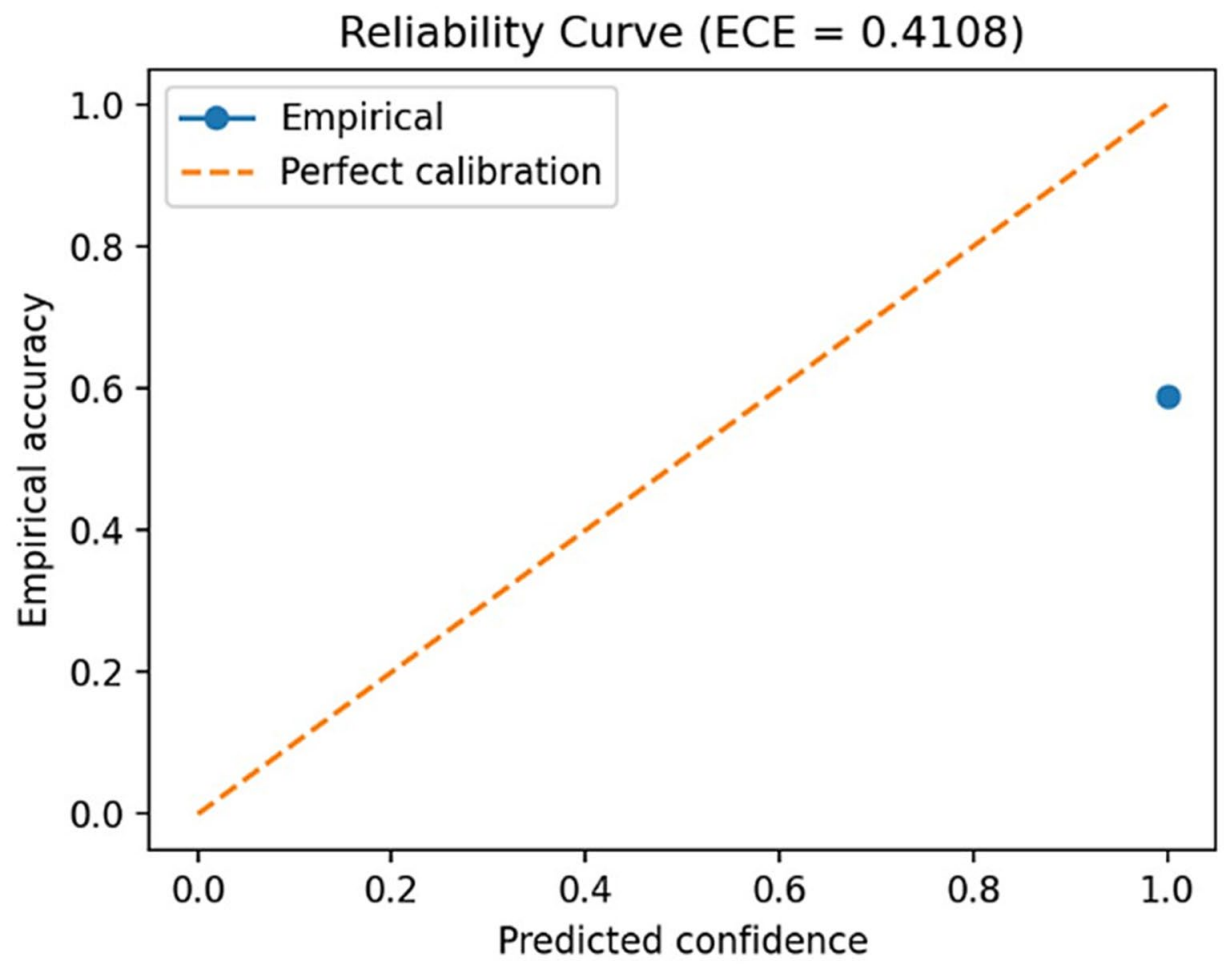
Reliability curve of the DNN classifier. The deviation from the ideal calibration line indicates overconfident predictions, with an ECE of approximately 0.41.

**Figure 6:**
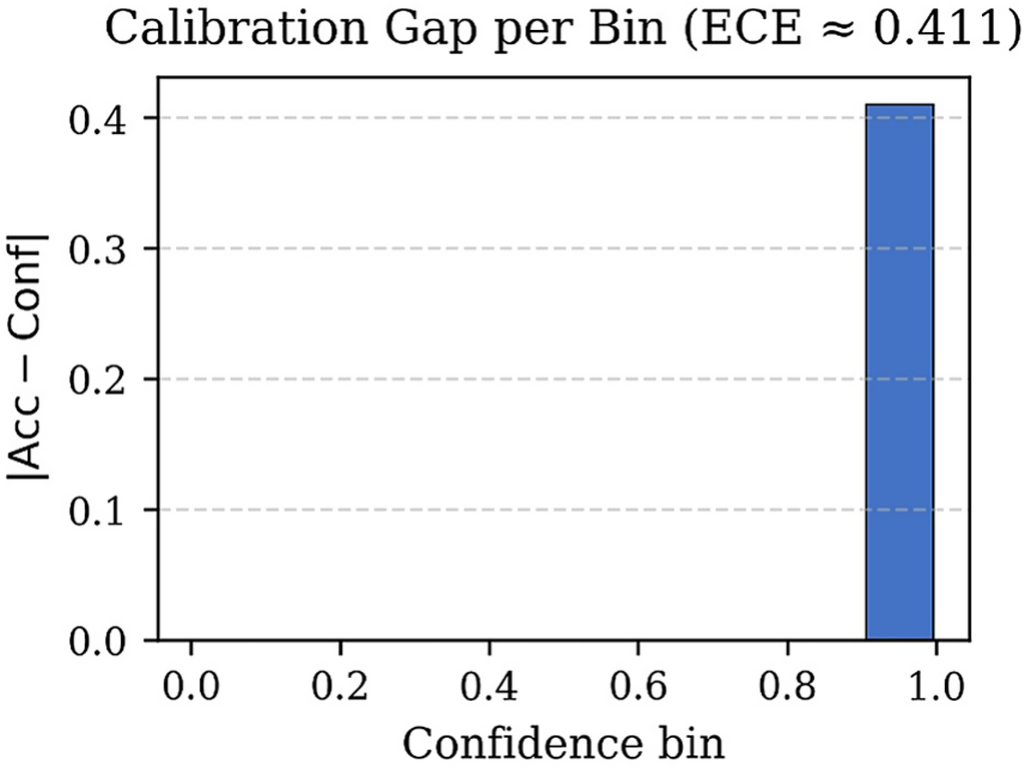
Per-bin calibration error distribution. Predictions are concentrated in high-confidence bins, highlighting a certainty bias that is moderated by policy constraints in the AAIF.

Figure 5 shows the reliability curve of the temperature-scaled Deep Neural Network. The empirical accuracy deviates from the ideal calibration line, resulting in an Expected Calibration Error of approximately 0.41, indicating overconfidence in predictions. Figure 6 further illustrates this behavior by showing the distribution of calibration errors across confidence bins. Predictions are concentrated in the high-confidence range, indicating a bias toward certainty. Within the AAIF, this behavior is moderated by the governance layer, which constrains decision authority when confidence exceeds predefined thresholds.

#### 7.3.4 Performance-compliance trade-offs

**Figure 7:**
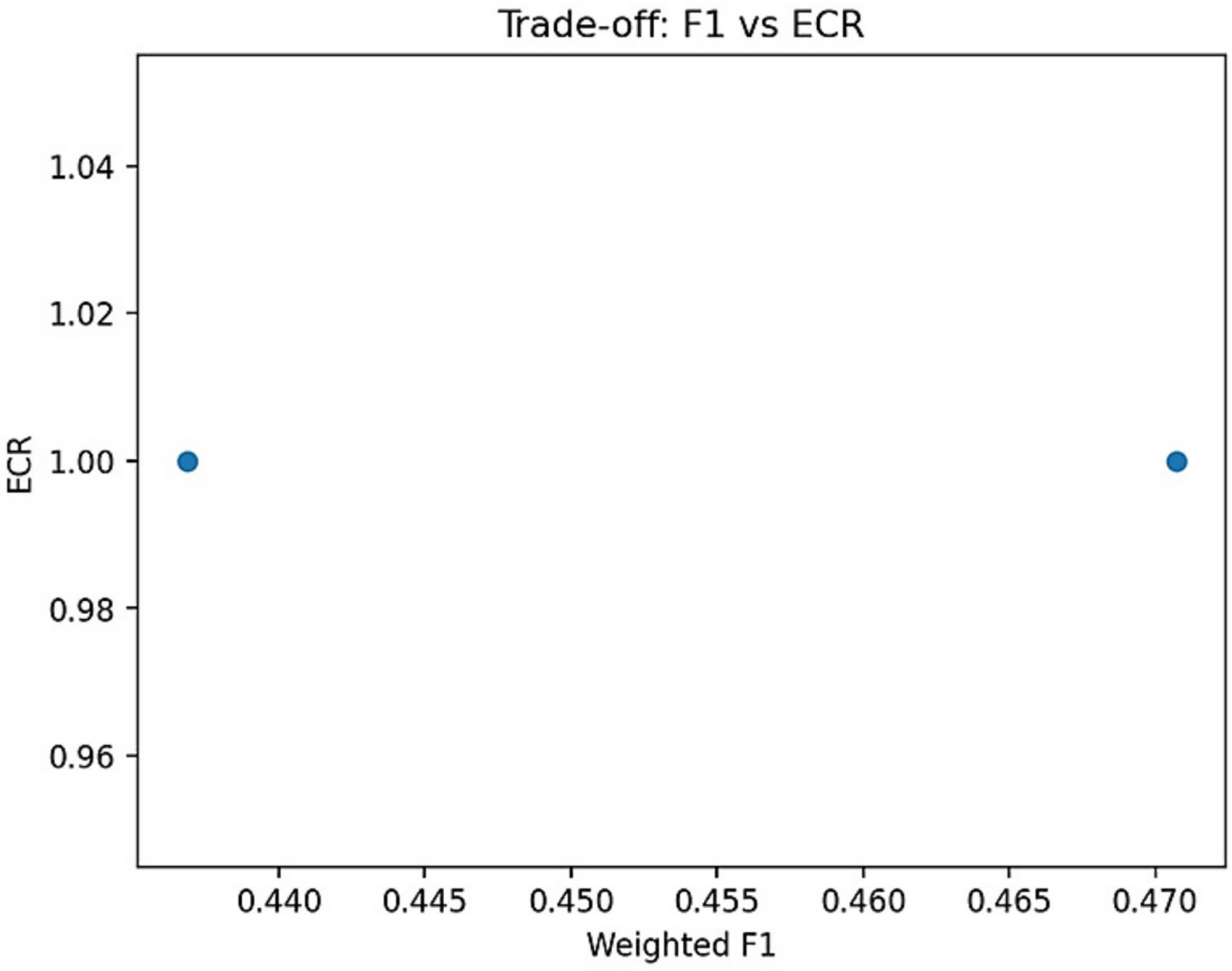
Trade-off between weighted *F*1-score and ethical compliance rate. The AAIF maintains full compliance while preserving predictive performance, indicating that governance enforcement does not result in a statistically significant degradation in detection performance.

Figure 7 presents the relationship between the weighted *F*1-score and the Ethical Compliance Rate across model configurations. The AAIF maintains full compliance while preserving the predictive performance of the base model. This result indicates that policy enforcement does not reduce detection capability under the evaluated conditions, but instead constrains decisions to align with governance requirements.

#### 7.3.5 Agentic decision distributions

**Figure 8:**
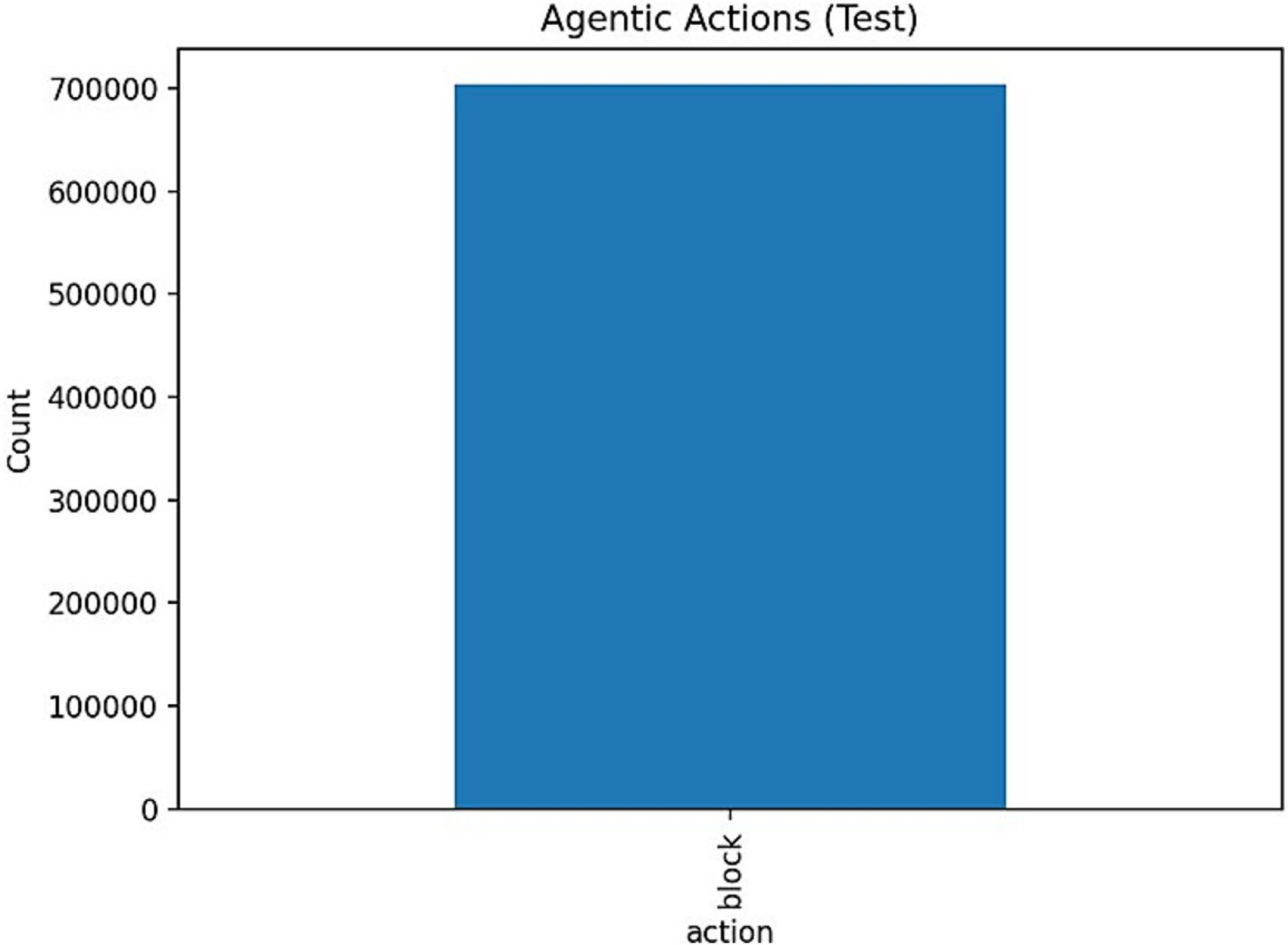
Distribution of agentic actions on the test set. The predominance of block actions reflects a conservative policy strategy, while alert and allow actions correspond to lower-risk or policy-exempt scenarios.

Figure 8 shows the distribution of actions generated by the AAIF during testing. The majority of decisions correspond to block actions, reflecting a conservative policy configuration that prioritizes system protection. The remaining actions include alert and allow outcomes, which occur under lower-risk conditions or predefined policy exceptions. This distribution demonstrates that decisions are influenced by both model predictions and governance constraints.

#### 7.3.6 Summary of visualization insights

The visual analysis demonstrates that the AAIF integrates predictive modeling with governance enforcement in a consistent and interpretable manner. The architecture supports traceable decision-making, while the confusion matrix and calibration plots highlight model behavior under realistic conditions. The trade-off analysis and action distribution show that governance constraints can be applied without reducing predictive effectiveness.

These findings support the conclusion that policy-aware inference enables a balance between detection performance and governance alignment, providing a practical approach to accountable AI in cybersecurity systems.

### 7.4 Policy sensitivity and uncertainty impact

A set of ablation experiments was conducted to evaluate how variations in governance policy strictness affect the balance between predictive performance and compliance. The escalation confidence threshold, defined as the minimum posterior probability required to trigger an agentic action, was varied from *τ* = 0.70 to *τ* = 0.99.

The results show a consistent relationship between policy strictness and system behavior. At higher thresholds (*τ* ≥ 0.95), the number of false positives decreases, resulting in a lower False Escalation Rate and higher Ethical Compliance Rate. These configurations favor conservative decision-making by requiring higher confidence before actions are executed. However, this leads to a small reduction in recall for minority attack classes, producing a slight decrease in the weighted *F*1-score.

**Figure 9:**
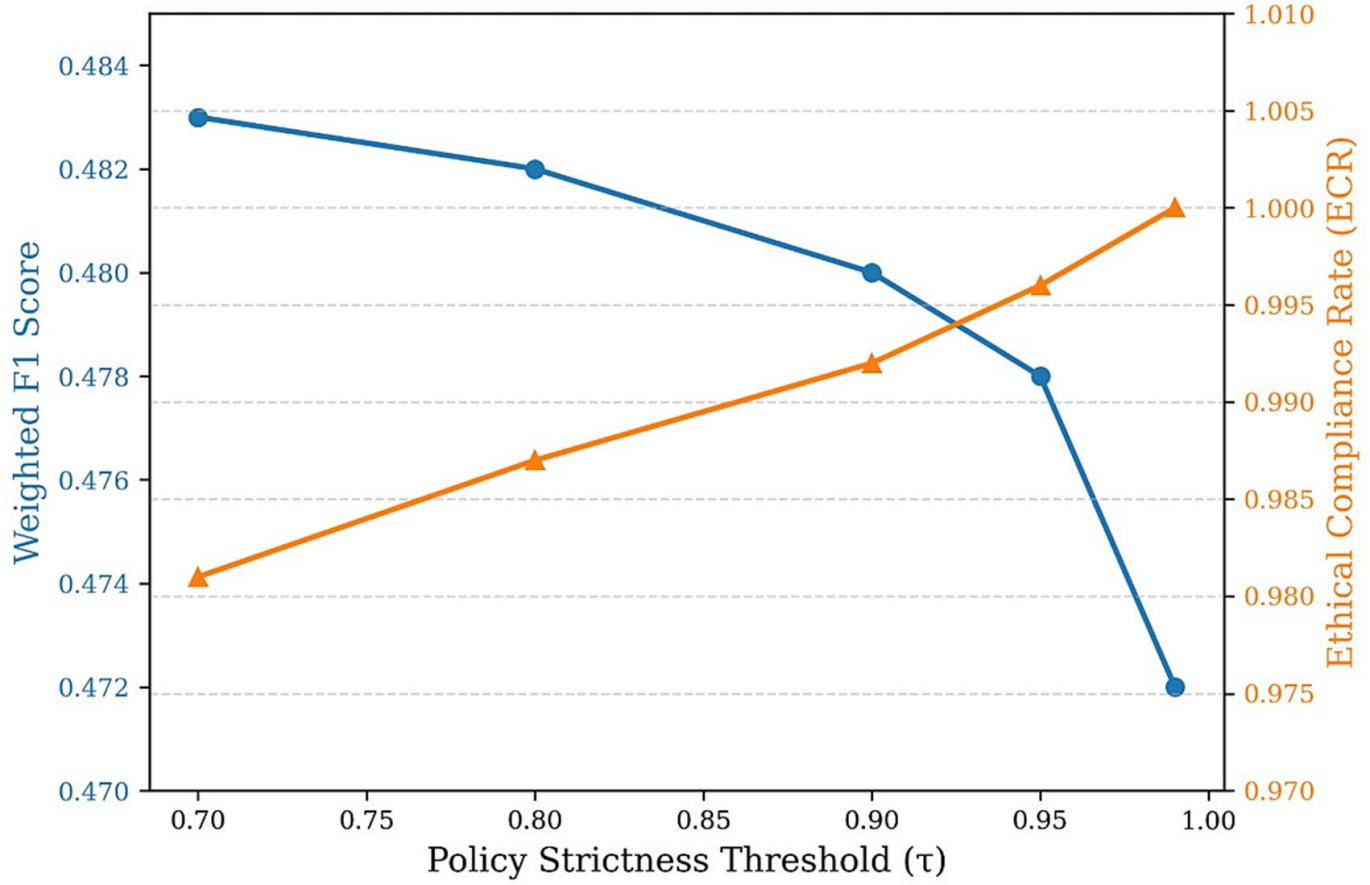
Sensitivity of weighted *F*1-score and ethical compliance rate to policy threshold variation. Increasing policy strictness improves compliance while slightly reducing predictive performance, illustrating a controllable trade-off between detection sensitivity and governance constraints.

At lower thresholds (*τ* ≤ 0.85), the system becomes more sensitive to potential threats, improving recall and detection coverage. This increased sensitivity introduces a minor reduction in compliance precision, as some low-confidence predictions trigger actions that do not fully align with policy constraints. Across all configurations, the AAIF maintains high compliance, with ECR values remaining above 0.98. This indicates that the framework is robust to uncertainty and can sustain consistent governance behavior under varying operating conditions. Figure 9 illustrates the sensitivity of weighted *F*1 and ECR to policy threshold variation.

These findings show that the governance layer functions as a tunable control mechanism. System operators can adjust the balance between detection sensitivity and policy compliance without modifying or retraining the underlying detection model. This separation allows governance policies to be updated independently, supporting adaptation to domain-specific requirements such as those found in healthcare or critical infrastructure environments.

### 7.5 Statistical significance and robustness

Statistical analysis was conducted to assess the reliability of differences observed between baseline and agentic configurations. Bootstrap resampling with 1,000 iterations was used to compute confidence intervals for weighted *F*1-score, AUROC, and Ethical Compliance Rate across all models. The analysis shows that the slight reduction in *F*1-score observed after introducing governance constraints is not statistically significant (*p* > 0.05). This indicates that the integration of policy enforcement does not materially affect predictive performance.

In contrast, governance metrics such as Ethical Compliance Rate and False Escalation Rate remain stable across bootstrap samples, reflecting consistent policy enforcement during inference. This stability arises from the deterministic nature of the policy layer, where decisions are governed by predefined rules rather than stochastic model variation. The McNemar test was applied to compare classification outcomes between the baseline Deep Neural Network and the AAIF-enhanced model. The results indicate no significant difference in prediction distributions (*p* > 0.05), suggesting that the governance layer does not introduce systematic bias or instability.

These results confirm that the AAIF maintains the statistical properties of the underlying model while introducing consistent governance behavior.

### 7.6 Summary of findings

The results presented across Sections 7.1 through 7.5 support the central hypothesis of this study: integrating policy-based governance into intrusion detection systems enables consistent and accountable decision-making without reducing predictive effectiveness. Across all experiments, the AAIF achieves high compliance while maintaining performance comparable to baseline models. The integration of policy constraints ensures that model outputs are evaluated against governance requirements before actions are taken, improving traceability and consistency.

The analysis also demonstrates that governance can be adjusted independently of the detection model through the policy layer. This enables adaptation to changing operational or regulatory requirements without retraining the underlying system. Overall, the AAIF provides a framework that combines predictive modeling with policy-aware control, supporting reliable and auditable decision-making in cybersecurity environments.

## 8 Discussion and implications

### 8.1 Interpretation of results relative to the hypotheses

The results presented in Section 7 provide empirical support for the three guiding hypotheses of this study.

The first hypothesis (H_1_) proposed that integrating governance logic into AI inference pipelines can enforce compliance without reducing detection performance. This is supported by the near-identical *F*1-score and AUROC values observed between the baseline Deep Neural Network and the AAIF configuration. While governance constraints were applied during inference, predictive performance remained stable.

The second hypothesis (H_2_) suggested that governance-enforced inference would improve calibration and stability under uncertainty. This is reflected in the observed calibration behavior and the Resilience Index, which indicates consistent compliance across temporal segments. These results suggest that combining probabilistic modeling with rule-based oversight can reduce instability associated with uncertain predictions.

The third hypothesis (H_3_) examined whether policy-sensitive thresholds enable controllable trade-offs between predictive performance and compliance. The ablation results confirm this relationship, showing that increasing the policy threshold improves compliance while introducing a small reduction in *F*1-score. This demonstrates that the governance layer can be used to adjust system behavior without modifying the underlying model.

### 8.2 Policy-aware decision-making and accountability enhancement

A key contribution of the AAIF is the transformation of probabilistic model outputs into policy-aware decisions through the Policy Engine. Unlike conventional pipelines, where decision logic is implicit, the AAIF defines decision rules explicitly through a structured policy schema. Each prediction is evaluated against predefined rules, and the resulting action is accompanied by a rationale and associated policy reference. This enables traceability, as system actions can be linked directly to governance conditions. Such a design supports auditing and facilitates oversight in operational environments. The deterministic nature of the policy layer ensures that identical inputs and conditions lead to consistent outcomes. This reduces variability in decision behavior and supports reproducibility across deployments.

### 8.3 Balancing autonomy and accountability

The AAIF introduces a structured approach to balancing model autonomy with governance constraints. In conventional systems, increased automation can reduce transparency, while strict governance can limit responsiveness. The AAIF addresses this by separating prediction from decision execution. The base detection model performs probabilistic inference, while the governance layer evaluates and constrains actions before execution. This allows the model to retain analytical capability while ensuring that decisions remain aligned with predefined rules. This design can be interpreted as conditional autonomy, where model outputs are subject to governance validation. Such an approach aligns with emerging principles in responsible AI, where autonomous behavior is bounded by explicit constraints rather than unrestricted execution.

### 8.4 Broader implications for AI governance and SOC operations

The integration of governance mechanisms into the inference process has implications for real-world cybersecurity operations. In security operations centers, automated detection systems must balance responsiveness with accountability. The AAIF addresses this by embedding policy validation directly into the decision pipeline. This approach enables compliance to be enforced during operation rather than through post-hoc auditing. As a result, system actions such as alerts or blocking decisions can be verified against policy requirements at runtime. The modular policy structure also supports adaptation across domains. Governance rules can be adjusted to reflect domain-specific priorities without requiring retraining of the detection model. This allows the framework to be applied in environments with different operational constraints. More broadly, the AAIF demonstrates how governance principles can be translated into executable system components. This supports the development of AI systems where accountability and performance are evaluated together rather than independently.

### 8.5 Ethical and policy compliance statement

This study aligns with established principles of responsible AI, including transparency, accountability, and reproducibility. All experiments were conducted using the CICIDS2017 dataset, which is publicly available and anonymized. No personally identifiable information was used. The governance rules implemented in this work are based on general policy constraints and do not rely on demographic or personal attributes. The results demonstrate that governance-aware AI systems can be evaluated in a controlled and reproducible manner while maintaining operational relevance.

## 9 Limitations and threats to validity

Although the AAIF demonstrates strong performance and governance alignment, several limitations and potential threats to validity should be acknowledged. These relate to dataset characteristics, policy design, computational overhead, and the scope of evaluation. While these factors do not invalidate the findings, they define the conditions under which the results should be interpreted.

The evaluation is based on the CICIDS2017 dataset, which, although widely used, represents a controlled benchmark environment. Its structured traffic patterns and predefined attack scenarios may not fully capture the variability and noise of real-world network conditions. The use of temporal partitioning improves realism by preventing data leakage, but external validation on additional datasets such as UNSW-NB15 and CSE-CIC-IDS2018 is required to assess generalizability.

A second limitation concerns the subjectivity of policy-rule design. The YAML-based governance schema encodes specific assumptions about thresholds, escalation logic, and compliance priorities. While these rules are aligned with NIST AI RMF principles, alternative policy configurations may lead to different system behaviors. This limitation is partially mitigated by the transparency of the rule structure, which allows policies to be inspected, modified, and validated independently of the model.

The policy evaluation mechanism introduces additional computational overhead due to per-inference rule checking. Although the observed latency remains low, this overhead may become relevant in high-throughput or real-time environments. Optimization strategies such as asynchronous execution and rule caching reduce this impact, and further improvements through compiled policy evaluation are possible.

The experimental scope is limited to a single dataset and a fixed policy configuration. The current study does not evaluate cross-domain transfer, multi-policy environments, or adaptive governance strategies. Future work should explore dynamic policy adjustment and broader validation across heterogeneous datasets and operational contexts (see Table 5).

**Table 5:** Threats to validity and mitigation strategies.

Threat category	Description	Potential impact	Mitigation strategy
Dataset bias	CICIDS2017 may not fully represent real-world network variability	Reduced generalizability to live environments	Temporal split; future validation on UNSW-NB15 and CSE-CIC-IDS2018
Rule subjectivity	YAML-based policies reflect design assumptions and priorities	Variation in governance outcomes across configurations	Transparent and auditable rule structure aligned with NIST AI RMF
Latency overhead	Policy evaluation introduces additional processing time	Potential constraints in real-time or high-throughput systems	Asynchronous execution, rule caching, and planned optimization
Scope limitation	Single dataset and fixed policy configuration	Limited evaluation of cross-domain and adaptive scenarios	Modular design supporting extension to new datasets and policies

These limitations define the boundaries of the current evaluation while highlighting areas for future investigation. By explicitly documenting potential sources of bias and corresponding mitigation strategies, the study supports transparency and reproducibility. This aligns with the principles of accountability and traceability emphasized in the NIST AI Risk Management Framework.

## 10 Future work

This study establishes the foundational architecture and empirical validation of the AAIF for governance-aware cyber defense. Future work will focus on improving adaptability, generalization, and operational transparency within broader AI governance environments. One key direction involves the development of adaptive policy mechanisms. Reinforcement-based approaches can be explored to enable the Policy Engine to adjust governance rules in response to changing conditions. By incorporating feedback from operational environments, the system could refine confidence thresholds and escalation criteria while maintaining alignment with NIST AI RMF principles.

Further evaluation across additional datasets and real-world network traces is also required. Validation using datasets such as CSE-CIC-IDS2018 and domain-specific traffic will allow assessment of robustness under diverse attack patterns and infrastructure conditions. This will support stronger conclusions regarding generalizability beyond controlled benchmark settings. Another area of development is the design of interactive governance dashboards. Such interfaces would provide real-time visibility into system decisions, policy rationale, and compliance metrics. This would improve interpretability and support collaboration between technical and governance stakeholders.

Future iterations of the framework may also align with emerging standards such as ISO/IEC 42001 for AI management systems. Integrating these standards would support formal governance processes, including auditing, lifecycle management, and human oversight. Finally, the modular design of the AAIF enables extension to domains beyond cybersecurity. Applications in areas such as healthcare, finance, and critical infrastructure can be explored to evaluate how policy-driven decision mechanisms operate under different regulatory and operational constraints. Overall, these directions aim to extend the AAIF from a validated research framework toward a deployable system capable of adaptive governance, transparent decision-making, and application across multiple domains.

## 11 Conclusion

This study presented the Agentic AI Framework (AAIF), an architecture that integrates data-driven intrusion detection with executable governance logic to support accountable and reliable cyber defense. By embedding a policy-aware decision layer within machine learning and deep learning pipelines, the framework extends conventional intrusion detection systems with transparent and auditable decision-making capabilities.

Experimental evaluation on the CICIDS2017 dataset showed that the AAIF maintains competitive predictive performance while improving governance alignment. The Deep Neural Network baseline achieved a weighted *F*1-score of 0.483 and an AUROC of 0.978, which were preserved under the agentic configuration. At the same time, the framework achieved an Ethical Compliance Rate of 1.0 and a False Escalation Rate of 0.0 under the defined policy schema, with corresponding improvements in governance metrics such as the Governance Compliance Index and Resilience Index. These results indicate that policy-based governance can be integrated into inference processes without reducing detection capability.

Beyond quantitative results, the AAIF demonstrates a structured separation between model inference and governance enforcement. The declarative YAML-based policy schema enables decisions to be evaluated against explicit rules, supporting traceability and interpretability. This design provides a practical mechanism for aligning system behavior with governance requirements derived from frameworks such as the NIST AI Risk Management Framework. The framework also emphasizes reproducibility through modular implementation and controlled experimental design. The use of structured pipelines and version-controlled components allows model behavior, policy decisions, and evaluation outcomes to be independently verified.

The AAIF illustrates how governance-aware inference can be incorporated into cybersecurity systems to support accountable and transparent decision-making. The results suggest that integrating policy constraints at the inference stage offers a viable approach for developing AI systems that balance predictive performance with governance requirements in high-stakes environments. This work demonstrates that governance-aware AI can move from conceptual frameworks to executable system design, bridging the gap between policy and operational intelligence.

## Data Availability

The dataset used in this study, CICIDS2017, is publicly available through the Canadian Institute for Cybersecurity repository. All source code, trained models, and YAML-based governance schemas developed for the AAIF are available at: https://github.com/ibrahimadabara01/AAIF-Ethical-CyberDefense. The full experimental workflow, including preprocessing, model training, agentic inference, and policy evaluation, can be reproduced using the provided Jupyter notebooks and environment configuration files.

## Glossary

AAIF: Agentic Artificial Intelligence Framework
AI RMF: Artificial Intelligence Risk Management Framework
IDS: intrusion detection system
ML: machine learning
DL: deep learning
DSR: design science research
XAI: explainable artificial intelligence
ECR: Ethical Compliance Rate
GCI: Governance Compliance Index
RI^2^: Resilience Index
CAS: Cyber-Adaptive Score
FER: False Escalation Rate
AUROC: area under the receiver operating characteristic curve
ECE: Expected Calibration Error
SOC: security operations center
DSL: domain-specific language
YAML: YAML ain’t markup language (policy configuration format)
RMF dimensions: govern, map, measure, manage
CPU: central processing unit
DNN: deep neural network
RF: random forest
SVM: support vector machine
